# Neural substrates associated with the acquisition of fine finger force control

**DOI:** 10.1186/s12868-025-00986-0

**Published:** 2025-12-13

**Authors:** Aoki Takahashi, Riku Ishizaka, Kodai Minami, Yuki Tanaka, Taisei Miyazaki, Kenta Oguma, Nodoka Shimizume, Tatsunori Watanabe

**Affiliations:** 1 Graduate School of Health Sciences, Aomori University of Health and Welfare, 58-1 Mase, Hamadate, Aomori, 030-8505 Japan; 2 Department of Rehabilitation Medicine, Tokyo Bay Rehabilitation Hospital, Chiba, Japan; 3 Waseda Institute for Sport Sciences, Waseda University, Tokorozawa, Saitama Japan

**Keywords:** Learning, Force control, Pinch grip, Cortex, Event-related spectral perturbations, Event related-potentials

## Abstract

**Background:**

Precise finger force control is essential for performing everyday tasks such as writing, buttoning, and eating. While the neurophysiological basis of cognitive skill learning has been extensively studied, much less is known about the neural mechanisms supporting the acquisition of fine motor skills involving finger force control. This study aimed to investigate changes in cortical oscillatory activity and event-related potentials (ERPs) associated with the acquisition of fine finger force control.

**Results:**

Eighteen right-handed healthy young adults practiced a visual target-matching task using a left-hand pinch grip. Force control performance and electroencephalogram (EEG) recordings were assessed before and after training. Behavioral analyses revealed significant improvements in time to target, velocity to target, mean force error, and force variability, with no changes in reaction time. EEG analysis showed enhanced alpha- and beta-band event-related desynchronization after learning. ERP analysis further revealed a significant reduction in N2 amplitude and a significant increase in P3 amplitude following learning.

**Conclusions:**

These results suggest that learning fine finger force control is accompanied by enhanced visuomotor processing, more efficient stimulus discrimination, and greater attentional allocation. This study provides novel insights into the neurophysiological underpinnings of fine motor skill acquisition.

## Background

Fine motor control of the fingers is essential for performing activities of daily living (ADLs), such as dressing, grooming, and eating. In particular, the ability to finely regulate muscular force output using visual information plays a crucial role in executing such dexterous movements. Therefore, when this ability is compromised by neurological disorders such as stroke [1, 2], which can cause a range of motor and sensory dysfunctions due to damage to cortical areas and neural pathways, facilitating the recovery of fine force regulation becomes a primary goal of rehabilitation. However, the neurophysiological mechanisms underlying improvements in force regulation remain inadequately understood.

Most previous studies on task learning have primarily focused on cognitive aspects, such as reaction time to visual stimuli [3] or the acquisition of sequential input patterns [4]. In contrast, relatively few studies have specifically examined the learning of force regulation. Notably, Gehringer et al. examined changes in brain activity associated with learning lower-limb force control tasks using magnetoencephalography in 2018 and 2019 [5, 6]. In both studies, healthy adults performed an isometric force regulation task involving ankle plantarflexion, guided by visual feedback presented on a screen. Participants were instructed to match their exerted force to a target that varied between 15% and 30% of their maximal voluntary isometric force across trials. In the 2018 study, motor performance improved over the course of learning, which was accompanied by a reduction in beta-band (15–30 Hz) event-related desynchronization (ERD) within the sensorimotor cortex [5], an index of cortical activity during motor planning and execution [7, 8]. Conversely, the 2019 study reported an enhancement of beta-band ERD in the sensorimotor cortex, despite comparable improvements in motor performance [6]. These contradictory findings, despite the use of identical tasks, underscore the lack of consensus regarding how ERD changes during the learning of force control tasks.

In addition to brain oscillations, several previous studies have investigated event-related potentials (ERPs) in relation to cognitive task leaning [3]. The frontal N2 component, in particular, is regarded as an index of conflict monitoring and stimulus evaluation and has been consistently associated with the recognition of novel visual stimuli [9, 10]. Importantly, its amplitude is highly sensitive to stimulus novelty [11, 12], reflecting novelty detection and the identification of mismatches relative to previously encountered stimuli. The parietal P3 component, on the other hand, reflects the allocation of attentional resources relevant to task performance [13, 14]. For example, Kóbor et al. compared the P3 component between statistical learning and sequence learning, reporting a reduction in P3 amplitude over time during sequence learning but not during statistical learning [15]. However, how these ERP components change during the acquisition of fine force control remains unclear.

The objective of the present study was to investigate how cortical responses, specifically the alpha- and beta-band ERD, as well as the N2 and P3 components of ERPs, change over the course of acquiring fine force control. Given that alpha- and beta-band ERD increases when precise force control is required [6, 16–19], we hypothesize that ERD would be enhanced following task learning. Also, since the N2 and P3 amplitudes are thought to reflect stimulus novelty and the allocation of attentional resources, respectively [15, 20, 21], we predicted that both components would show reduced amplitudes after learning.

## Materials and methods

### Participants

Eighteen healthy young adults (*M* = 21.9 years, *SD* = 1.7; eight females) participated in this study. All participants had normal or corrected-to-normal vision and were right-handed, as assessed by the Edinburgh Handedness Inventory (*M* = 94.7, *SD* = 9.1; range: 77.8–100) [22]. None of the participants had a history of neurological, psychological, cognitive, orthopedic, or cardiopulmonary disorders. The study procedure was explained to all participants prior to the experiment, and written informed consent was obtained from each participant. The study was approved by the Ethics Committee of Aomori University of Health and Welfare (23038) and was conducted in accordance with the Declaration of Helsinki (Clinical trial number: not applicable).

### Visual stimuli

**Fig. 1:**
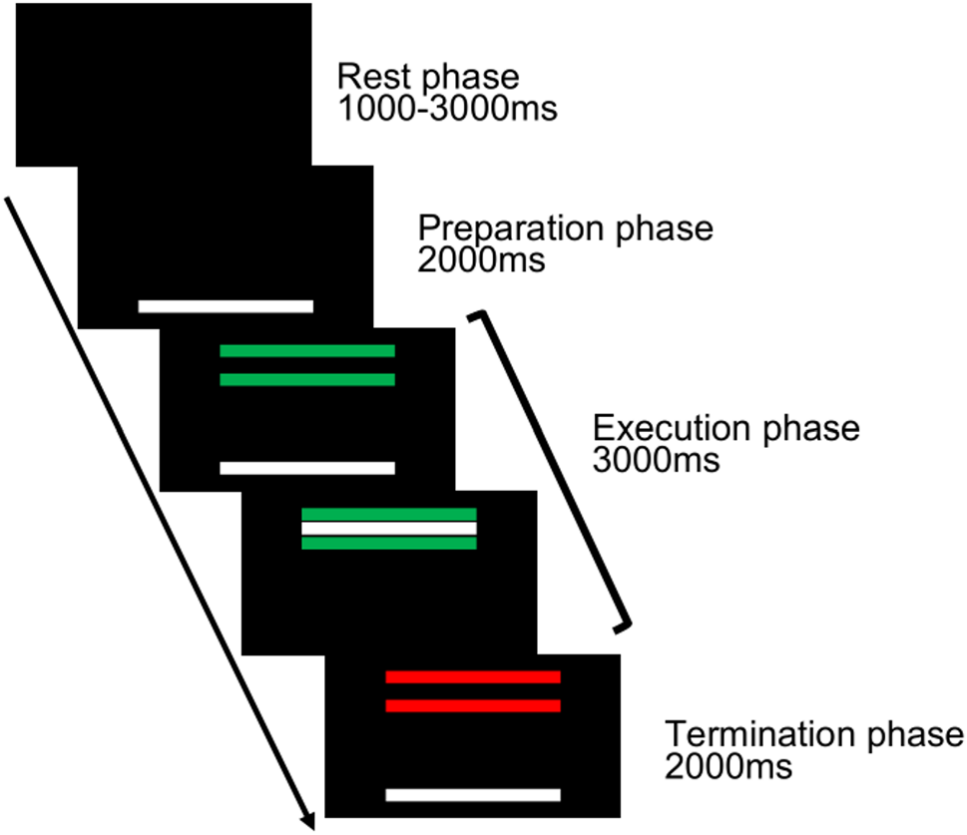
Visual stimuli in the target-matching task. Participants performed 450 trials of a left-hand pinch-grip visual target-matching task. Each trial began with a rest phase (1000–3000 ms), followed by a preparation phase (2000 ms), during which a white bar, moving vertically according to the applied force, appeared at the bottom of the screen. The execution phase started when two green parallel target bars appeared (3000 ms). Participants were instructed to exert force as quickly as possible and match the white bar within the target bars as accurately as possible. In the termination phase (2000 ms), the bars turned red, signaling participants to relax immediately. The black arrow on the left side indicates the sequence of screens

Visual stimuli were presented against a black background on a PC monitor (32GK850F-B, LG, Seoul, South Korea) placed 1 m in front of the participants at eye level, using a custom LabVIEW program (National Instruments, Austin, TX, USA). Each trial consisted of four phases: rest, preparation, execution, and termination (Fig. 1). The trial began with a rest phase lasting a random duration between 1000 and 3000 ms, during which no stimuli were presented and participants were instructed to relax. Following the rest phase, a white horizontal bar (force bar) indicating the force exerted by the participants was displayed for 2000 ms (preparation phase), during which participants were asked to prepare to exert force. This was followed by a 3000 ms execution phase, during which two green parallel bars (target bars) appeared above the force bar. The gap between the green bars was fixed and just wide enough to encompass the force bar. During this phase, the participants were instructed to exert force as quickly as possible and match the force within the target bars as accurately as possible. The final 2000 ms of the trial constituted the termination phase, during which the target bars turned red, and participants were instructed to relax immediately upon the color change.

The target bars were randomly positioned across trials at five force levels—10%, 12.5%, 15%, 17.5%, and 20% of each participant’s maximum voluntary isometric force (MVF). These levels were chosen to minimize potential fatigue effects during the task. Each force level was presented in 20% of the trials, ensuring an equal distribution across levels. The exerted force was low-pass filtered at 20 Hz, converted into Newtons, and digitized at 1 kHz using an A/D converter (National Instruments, Austin, TX, USA).

### Procedure

Participants were seated comfortably in a chair facing a PC monitor. They were instructed to hold a force transducer (Tech Gihan, Kyoto, Japan) and exerted a pinch-grip force using their left thumb and index finger. Prior to the experimental session, each participant’s MVF was determined. Participants were asked to gradually increase their pinch force over a period of 3 s and maintain the maximal force for an additional 2 s while receiving verbal encouragement. The highest force value obtained from three trials was recorded as the MVF.

Each participant completed a total of 450 trials divided into nine blocks of 50 trials each. To prevent fatigue, a minimum rest period of 3 min was provided between blocks. The first two blocks and the last two blocks were defined as the “pre-learning” condition and “post-learning” condition, respectively, while the middle five blocks served as practice blocks, similar to previous studies [5, 6].

### EEG recording

EEG data were recorded at a sampling rate of 1000 Hz with a band-pass filter of 0.01–200 Hz using a signal processor (Neuropack MEB-2300 system; Nihon-Kohden, Tokyo, Japan). Electrodes were placed at FCz, Cz, Pz, C3, and C4 according to the international 10–20 system. The ground electrode was placed on the forehead, and the reference electrode was attached to the right earlobe. Electrode impedance was maintained below 5 kΩ.

### Behavioral analysis

The force signals were low-pass filtered using a 4th-order Butterworth filter with a cutoff frequency of 15 Hz. Based on previous studies [5, 6], the following behavioral variables were calculated. Reaction time was defined as the interval between the onset of the execution phase and the time at which the force exceeded the mean ± 2 SD of the force values recorded during the 2-second preparation phase. Trials with reaction times shorter than 100 ms or longer than 1000 ms were excluded. On average, *M* = 7.7% (*SD* = 7.2) of trials per participant were excluded. Time to target was defined as the duration from reaction time to the time when the force bar entered the target range. Velocity to target was calculated by dividing the target force (%MVF) by the time to target. Mean force error (MFE) was defined as the average absolute difference between the target and exerted force (%MVF) during the final 2000 ms of the execution phase. Force variability was defined as the SD of the force during the final 2000 ms of the execution phase.

### Event related spectral perturbation analysis

EEG signals were processed using EEGLAB [23]. The data were filtered with a 1 Hz high-pass and 45 Hz low-pass filter and segmented into 5000 ms epochs beginning 1500 ms before the reaction time. Epochs exceeding ± 100 µV were removed. The number of remaining trials did not significantly differ between conditions (*p* >0.05; *M* = 92.1, *SE* = 2.0 for pre-learning, *M* = 90.4, *SE* = 1.4 for post-learning). Event-related spectral perturbation (ERSP) was calculated in the 3–45 Hz frequency range using Morlet wavelet transforms, with 3 cycles for the lowest and 9 cycles for the highest frequencies. ERSP values were computed using 200 time bins, yielding a temporal resolution of 30 ms. Values were baseline-normalized using the interval from − 1500 to -500 ms relative to the reaction time and expressed in decibels (dB). Since the focus was on alpha (8–14 Hz) and beta (15–30 Hz) band ERD during execution phase, analyses were conducted at the C3, Cz, and C4 electrodes.

### Event related potential analysis

As in ERSP analysis, EEG signals were processed using EEGLAB [23]. Data were filtered with a high-pass filter at 0.1 Hz and a low-pass filter at 40 Hz, then segmented into 1200 ms epochs starting 200 ms before the onset of the visual stimulus (i.e., the appearance of the white force bar in the preparation phase) (i.e., stimulus-locked). Epochs with EEG fluctuations exceeding ± 100 µV were excluded from analysis. The number of remaining trials did not differ significantly between conditions (*p* >0.05; *M* = 90.6, *SE* = 2.2 for pre-learning, *M* = 91.3, *SE* = 1.3 for post-learning). Baseline correction was performed using the mean voltage during the 200 ms preceding the visual stimulus. Analysis focused on the FCz and Pz electrodes, where the N2 and P3 components have been observed in previous studies [24].

### Statistical analysis

The normality of the behavioral variables (reaction time, time to target, velocity to target, MFE, and force variability) was assessed using the Shapiro–Wilk test. Paired t-tests were conducted to compare values between the pre-leaning and post-learning conditions. EEG data were analyzed using bootstrap paired t-tests implemented in EEGLAB, comparing the pre- and post-learning conditions. Multiple comparisons were corrected using the false discovery rate (FDR) method, applied to the time–frequency distribution of p-values separately for each channel. A significance level of 0.05 was adopted for all statistical tests.

## Results

### Behavior

**Fig. 2:**
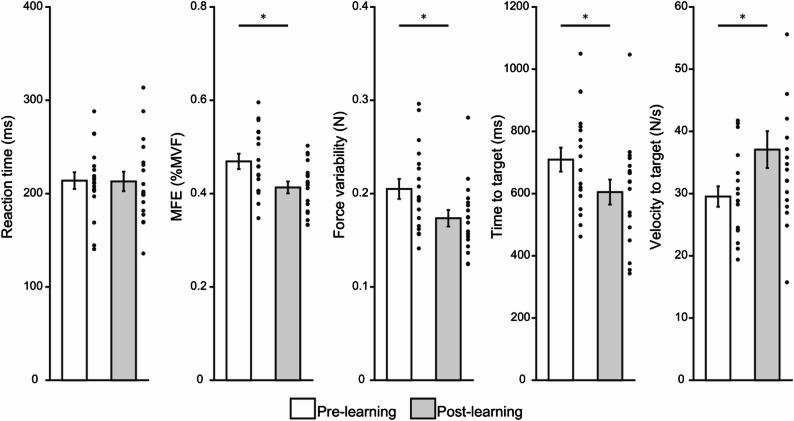
Behavioral variables before and after task learning. White bars represent pre-learning conditions, and gray bars represent post-learning conditions. Error bars indicate the standard error of the mean, and black dots indicate individual data points. Asterisks denote statistically significant differences (*p* < 0.05). MFE, mean force error; MVF, maximum voluntary force

Figure 2 presents the results of paired *t*-tests conducted on five behavioral indices before and after task learning. Reaction time did not differ significantly between the pre-learning (*M* = 213.9 ms, *SD* = 37.8) and post-learning (*M* = 213.0 ms, *SD* = 44.2) conditions (*t*(17) = 0.07, *p* > 0.05, Cohen`s *d* = 0.01). In contrast, several other indices showed significant improvement following learning. Specifically, time to target decreased significantly from *M* = 709.2 ms (*SD* = 163.0) to *M* = 604.8 ms (*SD* = 169.7) (*t*(17) = 2.3, *p* = 0.029, Cohen’s *d* = 0.56). Similarly, velocity to target increased from *M* = 29.5 N/s (*SD* = 6.9) to *M* = 37.0 N/s (*SD* = 12.5) (*t*(17) = -3.7, *p* = 0.002, Cohen’s *d* = -0.88). MFE was reduced from *M* = 0.46 %MVF (*SD* = 0.06) to *M* = 0.41 %MVF (*SD* = 0.05) (*t*(17) = 2.8, *p* = 0.012, Cohen’s *d* = 0.66). Finally, force variability decreased from *M* = 0.20 N (*SD* = 0.04) to *M* = 0.17 N (*SD* = 0.03) (*t*(17) = 4.2, *p* = 0.001, Cohen’s *d* = 0.99).

### Event-related spectral perturbation

**Fig. 3:**
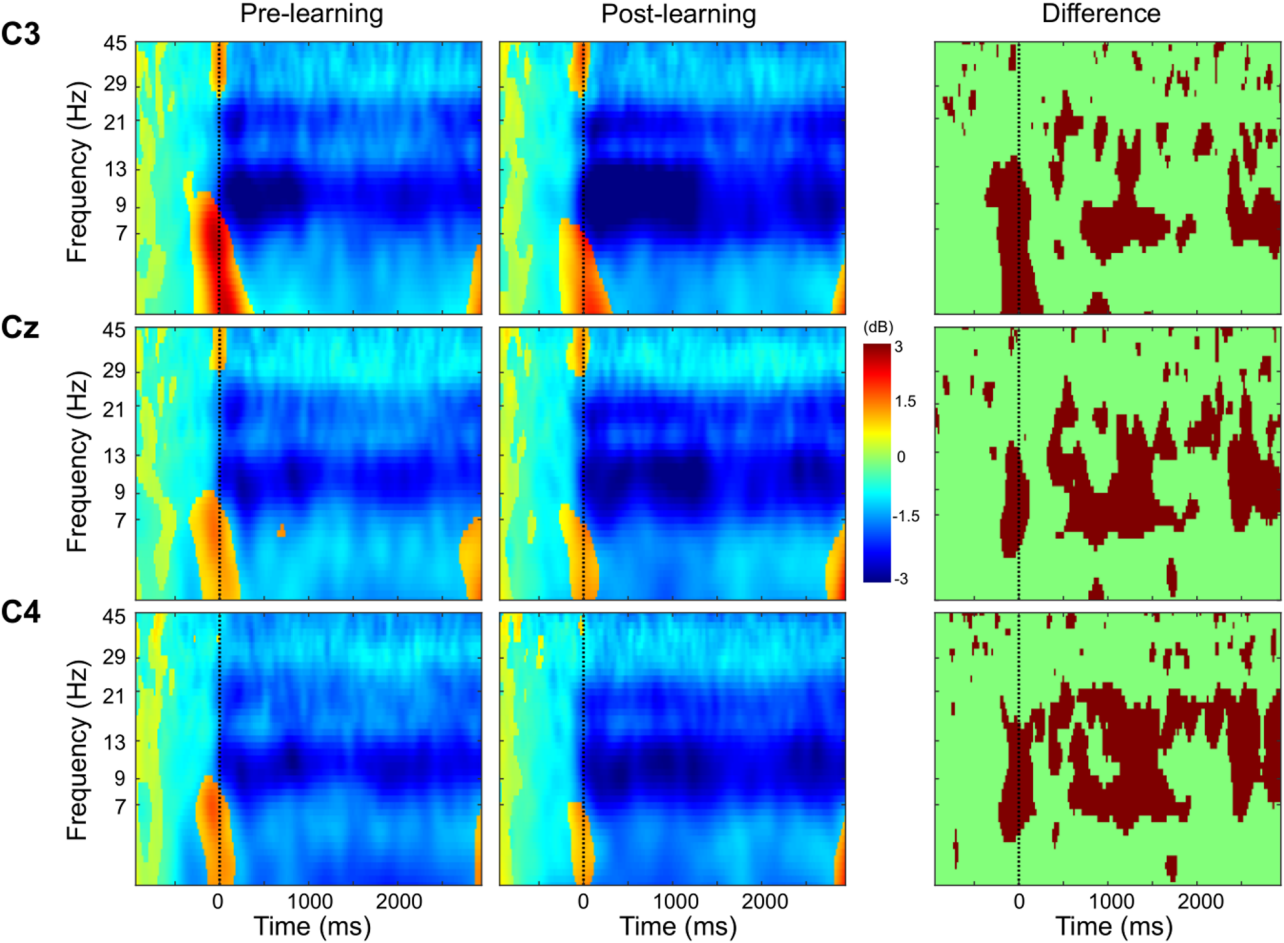
Grand-average event-related spectral perturbations at C3, Cz, and C4 electrodes. The color scale represents relative power changes from the baseline period (− 1500 to − 500 ms), expressed in decibels (dB), with red indicating positive changes and blue indicating negative changes. Time zero corresponds to the reaction time. The rightmost column for each electrode presents areas of significant differences between pre- and post-learning conditions (*p* < 0.05, FDR corrected)

Figure 3 shows the grand-average ERSP time-frequency plots recorded at C3, Cz, and C4 electrodes. ERD is depicted in blue (negative values), and event-related synchronization (ERS) is shown in red (positive values). ERD in the alpha and beta frequency bands was evident immediately following the reaction time at all three electrodes for both pre- and post-learning conditions. The plots to the right of each electrode illustrate regions showing significant differences between the two conditions. Regardless of the electrode, ERD in the alpha and beta bands was significantly greater in the post-learning condition compared to the pre-learning condition.

### Event-related potentials

**Fig. 4:**
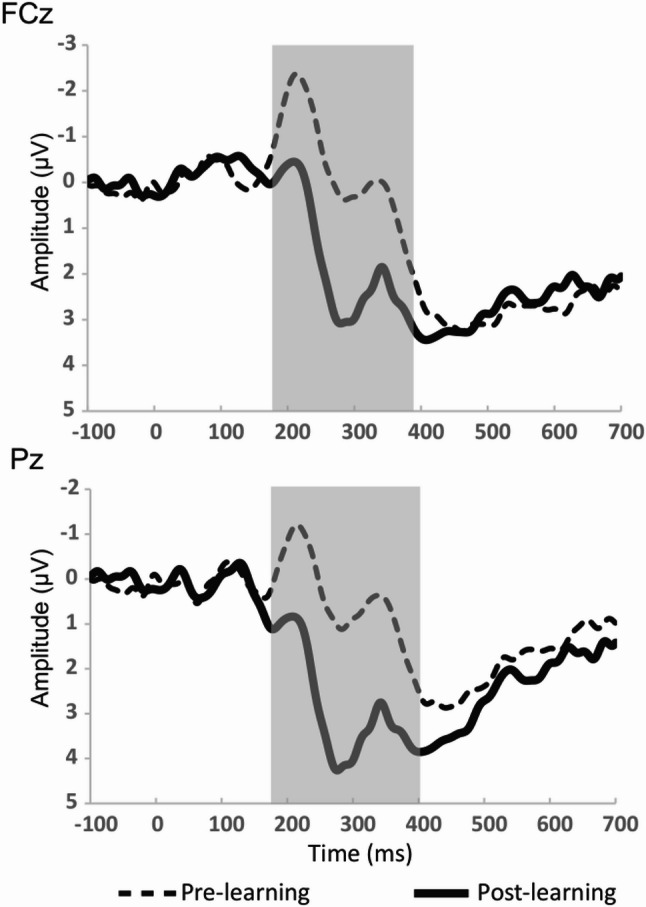
Grand-average event-related potentials at FCz and Pz electrodes. Dashed lines indicate waveforms recorded before learning, while solid lines indicate those recorded after learning. Shaded areas indicate significant differences between the pre- and post-learning conditions (*p* < 0.05, FDR corrected)

Figure 4 displays the grand-average ERP waveforms recorded at the FCz and Pz electrodes for the pre- and post-learning conditions. In both conditions, the ERP waveforms were characterized by the N2 and P3 components. Dashed lines represent the waveforms before learning, and solid lines those after learning. Gray shaded areas represent significant differences between conditions (*p* < 0.05). The N2 amplitude significantly reduced following task learning, whereas the P3 amplitude significantly increased.

## Discussion

In the present study, we examined how cortical responses, specifically alpha- and beta-band ERD, as well as the N2 and P3 components of ERPs, change following task learning in a force control paradigm. Based on previous research, we hypothesized that task learning would improve behavioral performance, accompanied by enhanced ERD and reduced N2 and P3 amplitudes. Consistent with this hypothesis, most behavioral performance indices improved following training, along with increased alpha- and beta-band ERD and a reduction in N2 amplitude. However, contrary to our expectations, the P3 component increased after learning. These findings provide new insights into the neural mechanisms underlying the acquisition of fine finger force control.

### Behavioral performance

Our behavioral results demonstrated significant improvements across multiple performance metrics following task learning. These findings are consistent with previous studies involving lower-limb tasks [5, 6]. The behavioral indices employed in this study are well-established measures of force control ability [25]. In particular, reductions in MFE and force variability have been reported in studies emphasizing precise motor adjustments facilitated by visual feedback [18, 26]. In line with these findings, our results suggest that participants acquired more accurate force regulation through learning.

### Event-related spectral perturbation

Alpha- and beta-band ERD refers to a relative decrease in power within these frequency bands compared to baseline levels [7]. Both alpha- and beta-band ERD similarly emerge over central areas before and during movement [8, 27, 28]. These oscillatory markers are closely associated with sensorimotor processing during task performance [29] and are thought to reflect increased cortical engagement in task-relevant neural networks [7, 8]. While alpha-band ERD tends to originate from postcentral sources and beta-band ERD from precentral sources during movement tasks [28, 30, 31], suggesting that alpha- and beta-band ERD may predominantly reflect somatosensory and motor processes, respectively, their functional differentiation, particularly regarding movement planning versus execution, remains to be fully elucidated. Recent studies have shown that alpha- and beta-band ERD change as motor learning progresses; however, there is currently no consensus regarding how movement-induced ERD is modulated during the learning of force regulation tasks. Two previous studies from the same research group investigated lower-limb isometric force tasks in healthy adults. One study, conducted with young adults (*M* = 23.3 years, *SD* = 3.3), reported a decrease in beta-band ERD with learning [5], whereas another study involving middle-aged adults (*M* = 36.6 years, *SD* = 5.0) and adolescents (*M* = 14.0 years, *SD* = 2.1) found an increase in adults and a decrease in adolescents [6]. The authors interpreted the reduced ERD as reflecting decreased demands on neural resources [5, 32], whereas the increased ERD was attributed to the use of familiar, everyday movements that may enhance sensorimotor cortex activation following learning [6]. Given that the mean age of participants in the present study (*M* = 21.9 years, *SD* = 1.7) was closer to that of the former study [5] or to adolescents [6], a reduction in ERD with learning might have been expected. Nevertheless, our results were more consistent with the latter study conducted in middle-aged adults [6], revealing not only increased beta-band ERD but also an additional enhancement of alpha-band ERD, even though the pinch grip task used in the present experiment does not appear to represent a familiar movement for participants. Notably, previous studies have shown that enhanced alpha- and beta-band ERD is associated with finer force regulation, especially reductions in MFE and force variability achieved by manipulating visual feedback to facilitate more precise control [16–19]. Therefore, it is plausible that the acquisition of precise force control, as reflected by reduced force error and variability, leads to stronger alpha- and beta-band ERD, possibly indicating efficient sensory and motor processing.

Although the precise mechanisms remain unclear, several factors may account for the discrepancy between our findings and those of the previous study reporting decreased ERD after learning [5]. First, the stage of learning may influence cortical activity, as suggested by meta-analyses comparing short- and long-term motor learning tasks (e.g., sensorimotor adaptation and serial reaction time tasks) [33]. For instance, Floyer-Lea et al. (2004) found decreased primary motor cortex activation following short-term learning (16 min) but increased activation after long-term learning (3 weeks of practice) [34]. In our study, the total training duration exceeded 1.5 h, whereas in the previous study it was estimated to be under 1 h [5]. Thus, differences in learning stage might have contributed to the divergent ERD patterns. However, as both studies conducted the task within a single day, interpreting these differences as reflecting short- versus long-term learning phases [33, 34] should be approached with caution. Second, the limb involved in the task (upper vs. lower limb) may have influenced the ERD outcomes. Indeed, a comparison between wrist extension and ankle dorsiflexion tasks demonstrated stronger alpha- and beta-band ERD during wrist movements, particularly in the preparation phase [35]. Moreover, using a power-grip force-tracking task with visual feedback that included both steady and ramp contractions, Kranczioch et al. (2008) found no significant changes in alpha- or beta-band ERD during the steady contraction phase, but observed a significant decrease in beta-band ERD over central regions during the ramp contraction phase after learning [36]. These findings suggest that not only the involved limb but also the type of contraction can influence learning-related changes in the ERD, highlighting the need for further investigation.

### Event-related potentials

Consistent with our initial hypothesis, we observed a reduction in N2 amplitude following learning of fine finger force control. The N2 amplitude has been associated with the recognition of novel or deviant visual stimuli [9, 10] and is known to be modulated by task learning. For example, Song et al. reported that N2 amplitude decreased over time as reaction times to target stimuli shortened in visual discrimination tasks [20]. Collectively, the observed reduction in N2 amplitude in the present study can be attributed to habituation to repeated visual stimuli and facilitated stimulus recognition due to increased familiarity. This finding extends previous research by demonstrating that N2 amplitude reductions through learning can occur not only in cognitive tasks but also in fine finger force control tasks.

In contrast to the N2 results, the findings for the P3 component did not support our initial hypothesis. The P3 is known to reflect the allocation of attentional resources [13, 14]. For instance, when a P3 is elicited by a secondary task or stimulus during performance of a primary task, its amplitude typically decreases as the primary task becomes more demanding, indicating fewer resources available for processing the secondary stimulus [37–39]. Similarly, P3 amplitude has been reported to decrease over the course of sequence learning [15], suggesting reduced attentional demands as tasks become more automated. Based on this evidence, we hypothesized that P3 amplitude would likewise decrease following the learning of fine finger force control. Contrary to expectations, however, we observed a significant increase in P3 amplitude, indicating that attentional demands may have risen rather than diminished after learning. Although this result is somewhat difficult to interpret, it may be explained by the characteristics of our experimental task. In the current force control task, the position of the target bars varied randomly across trials at five levels (10%, 12.5%, 15%, 17.5%, and 20% of each participant’s MVF) to increase task difficulty and simulate the variability encountered in activities of daily living. As a result, participants needed to pay close attention to the display to identify the target location and determine the required initial force on a trial-by-trial basis. Therefore, while the visual stimuli themselves may have become more familiar through learning, the demand for rapid and accurate responses to variable targets may have increased overall attentional load. Supporting this interpretation, a previous study comparing ERPs in response to simple and complex stimuli during visual perceptual learning found that P3 amplitude increased following learning with complex stimuli, but not with simple ones [21]. These findings suggest that the impact of task learning on P3 amplitude may depend on stimulus and/or task complexity. Future studies should investigate whether P3 amplitude decreases with extended training over longer timescales, potentially reflecting the transition to more automated performance.

### Limitations

Several limitations should be acknowledged. First, our findings may be specific to the task employed, as the same task was used for both the learning and post-learning evaluation phases. Future work should examine the generalizability by incorporating transfer tasks or clinically relevant assessments. Second, the present study investigated ERD and ERP based on recordings from a limited set of electrodes. Source-based analyses using high-density EEG to localize neural generators could have provided additional information about activity in both task-related and task-unrelated regions. Including a broader range of cortical areas would provide a more comprehensive understanding of the underlying neural mechanisms and should be considered in future research. Third, we did not evaluate the automatization of learning, a critical aspect of skill acquisition that involves shifts in neural engagement over time [33, 34]. Addressing automatization in future work will help clarify the neurophysiological processes associated with different stages of motor learning. Finally, EEG data were not recorded during the middle blocks of learning. Including these data would have provided valuable information on the sequential changes in brain activity.

## Conclusions

The acquisition of fine finger force control was associated with increased alpha- and beta-band ERD, reduced N2 amplitude, and increased P3 amplitude. These neural modulations likely reflect more efficient visuomotor processing, facilitated recognition of visual stimuli, and increased attentional demands. Together, these findings suggest that the neural correlates of learning fine finger force control may differ from those underlying general cognitive abilities and could depend on the limb involved in the task.

## Data Availability

The datasets used and/or analyzed during the current study are available from the corresponding author on reasonable request.
